# Spanish nurses’ experiences with personal protective equipment and perceptions of risk of contagion from COVID‐19: A qualitative rapid appraisal

**DOI:** 10.1111/jocn.16031

**Published:** 2021-09-15

**Authors:** Maria Romeu‐Labayen, Glòria Tort‐Nasarre, Bruna Alvarez, Martí Subias‐Miquel, Eva Vázquez‐Segura, Diana Marre, Paola Galbany‐Estragués

**Affiliations:** ^1^ AFIN Research Group and Outreach Centre Autonomous University of Barcelona Cerdanyola del Vallès Spain; ^2^ Department of Public Health, Mental Health and Mother‐Infant Nursing Faculty of Medicine and Health Sciences University of Barcelona L’Hospitalet del Llobregat Spain; ^3^ Department of Nursing Faculty of Nursing and Physiotherapy University of Lleida Lleida Spain; ^4^ Health Education Research Group (GREpS) Faculty of Nursing and Physiotherapy University of Lleida Lleida Spain; ^5^ Anoia Primary Care Service Gerència Territorial Catalunya Central Institut Català de la Salut (ICS) St. Fruitós del Bages Spain; ^6^ Parc Sanitari Sant Joan de Déu Sant Boi de Llobregat Spain; ^7^ Department of Public Health, Mental Health and Mother‐Infant Nursing Faculty of Medicine and Health Sciences University of Barcelona Barcelona Spain; ^8^ Manso’s Sexual and Reproductive Healthcare Center Gerència Territorial Barcelona Institut Català de la Salut (ICS) Barcelona Spain; ^9^ Research Group on Methodology, Methods, Models and Outcomes of Health and Social Sciences (M3O) Faculty of Health Science and Welfare Centre for Health and Social Care Research (CESS) University of Vic‐Central University of Catalonia (UVIC‐UCC) Vic Spain

**Keywords:** crisis intervention, health risk, practice nursing, qualitative approaches

## Abstract

**Aims and Objetives:**

Explore nurses' experiences and perception of risk regarding the use of personal protective equipment during the first wave of the pandemic in Spain.

**Background:**

The contribution of our study is to use qualitative methods to understand nurses' experiences and perceptions of the risk of the contagion linked to the shortage of PPE during the first wave of the pandemic, whose explosive start strained health systems around the globe.

**Design:**

Qualitative descriptive design according to the Rapid Research Evaluation and Appraisal model.

**Methods:**

Semi‐structured videoconference interviews were conducted to explore the experiences of 29 nurses including staff nurses, nursing supervisors and nursing directors from hospital and community services of the Spanish health system. Interviews lasted 30–45 min and were conducted in May 2020. We carried out a thematic analysis using Dedoose. The COREQ checklist was used to report findings.

**Results:**

We identified the following themes and subthemes: 1. Experiences with personal protective equipment: scarcity, inequality, reutilization, self‐protection, delegation of responsibility, and gap between protocols and reality; 2. Perception of the risk of contagion: lack of credibility, lack of trust, lack of support, and meeting subjective needs.

**Conclusions:**

The scarcity of personal protective equipment and inequality in its distribution led nurses to take initiatives to feel more protected. Mid‐ranking supervisors were caught between the responsibility of monitoring and rationing personal protective equipment and providing the necessary protection to nurses. The disjuncture between protocols and the available supply of personal protective equipment caused confusion. Lack of credibility, lack of trust and lack of support from management influenced participants' perception of the risk of contagion. Mid‐ranking supervisors were often responsible for trying to alleviate fear among nursing staff.

**Relevance to clinical practice:**

Understanding the factors involved in risk perception can be helpful to decision‐makers who help protect nurses in clinical practice. These results can help administrators and policymakers because they point to the need for nurses to feel that their departments and centers look after their safety at work. Transparent communication and emotional support may contribute to their well‐being in the face of risk.

**What does this paper contribute to the wider global clinical community?**

- The scarcity of personal protective equipment and inequality in its distribution led participants to take initiatives such as reusing equipment and constructing their own with non‐approved materials. Participants who were mid‐ranking supervisors monitored the use of personal protective equipment.
- Supervisors were caught between the responsibility of rationing personal protective equipment and that of providing the necessary protection to nurses. There was a gap between the protocols surrounding the use of PPE and the reality of the available equipment.
- Lack of credibility, lack of confidence and lack of support from management worsened the perception of risk of contagion of participants. Mid‐ranking supervisors became responsible for managing fear and the perception of risk among their nursing staff.

## 1 INTRODUCTION

The World Health Organization (WHO) declared the outbreak of COVID‐19 an international public health emergency on 30 January 2020 (Mahase, 2020). After the disease spread rapidly around the world, it was declared a pandemic on March 11, 2020 (WHO, 2020). Through Royal Decree 463/2020 of March 14, 2020, the Spanish government declared a state of alarm that included a stay‐at‐home order to prevent the spread of the disease (Ministerio de la Presidencia & Relaciones con las Cortes y Memoria Democrática, 2020). This was the start of an unprecedented public health crisis that has upset not only people’ lives but also the country’s social, economic, political and health structure.

## 2 BACKGROUND

The protection of health professionals during a pandemic is key to preventing community transmission (WHO, 2018). Protection materials and protocols have evolved since the 1980s, when the HIV‐AIDS pandemic began. In 1985, the Centers for Disease Control and Prevention introduced the ‘universal precautions’ to prevent contagion from bloodborne pathogens. In 1996, they were revised as the ‘standard precautions’, which defined protection against the main transmission pathways for infectious disease: aerosols, drops and contact (Wolf, 2005). The main measure is handwashing and the main physical barrier is personal protective equipment (PPE), which protects skin, mucous membranes, the respiratory tract and clothing. PPE includes gowns, gloves, masks, face shields and goggles (Wolf, 2005).

During the HIV‐AIDS pandemic, Gerbert et al. (1988) showed that nurses’ perception of risk was linked, on the one hand, to the fact that authority figures minimised the likelihood of infection and introduced protocols that didn't guarantee nurses’ safety and, on the other hand, to communication problems between nurses and health authorities, caused in part by nurses’ own subjective risk assessment. The authors noted that mistrust is one of the main engines of fear. Gallop et al. (1992) concluded that fear of contagion was a major concern for nurses who worked with HIV patients and that health administrators must comprehend the great complexity of risk perception in order to offer solutions.

More recently, the world has witnessed several pandemics caused by respiratory illnesses with airborne transmission. Severe Acute Respiratory Syndrome (SARS) in 2002 and Middle East Respiratory Syndrome (MERS) in 2012 affected Asia, the Middle East, Europe and the Americas (Høiby, 2020). In 2007, the WHO recommended reinforcing handwashing and the use of masks, glasses and gloves to prevent transmission between health professionals and patients and established the relationship between erratic use of PPE the transmission of nosocomial of avian flu virus (WHO, 2007). Corley et al. (2010) provided a qualitative description of the experiences of nurses who cared for people who were sick with or suspected of having H1N1 at a hospital in Queensland. Some participants felt unprotected because the protocols surrounding PPE were unclear, and sometimes they perceived that protocols were modified according to the availability of PPE instead of according to safety criteria. A qualitative study about the MERS pandemic in Korea concluded that the experience of nurses should be taken as a starting point for designing protection strategies for future epidemics or pandemics. The authors showed that nurses felt unprotected, alone, tired, and stigmatised. At the same time, feelings of comradery, the responsibility of caring for others, and the social support they received helped them grow as nurses and maintain their commitment to provide care despite the adversity that they faced (Kim, 2018).

The recent epidemics of Ebola virus disease (EVD) in Africa between 2014 and 2016 also provided a useful comparison to the COVID‐19 pandemic because the high transmissibility of the virus put health systems around the globe on alert. EVD’s transmission pathway is direct contact with fluids: blood, secretions and organs of infected people as well as contact with contaminated materials. Contact with persons with EVD carries a high risk of infection, which itself carries a high risk of death. Strict protocols for putting on and removing PPE were implemented. The lack of sufficient PPE and the improper removal of PPE carried high risk of contagion (Adongo et al., 2017; Reidy et al., 2017). In the United States, nurses preparing for the arrival of patients with EVD noted that the lack of a specific treatment, the scarcity of PPE and the lack of training about how to use PPE with these patients caused them to fear contagion (Gabel Speroni et al., 2015). In Australia, where nurses also prepared for patients with EVD (although none arrived), they relied on their institutions’ good organisation and preparation to address a possible EVD case. Under these circumstances, nurses’ perception of risk focused on the fear of infecting their families (Pincha Baduge et al., 2017).

Historically, nurses have played an important role in infection prevention, the monitoring of isolated patients, containment and public health (Smith et al., 2020). During the COVID‐19 pandemic, nurses have faced fear of contagion, an emotion shared by healthcare professionals around the world (Liu et al., 2020). COVID‐19 contagion among healthcare professionals has been linked to working at close range of patients without PPE and having had close contact with infected people (Jin et al., 2020). Infected healthcare professionals initially wore little protection because they were unaware of the transmission capacity of the SARS‐CoV‐2 virus and they also lacked information about whether their patients were infected (Wei et al., 2020).

In Spain, healthcare professionals had insufficient access to PPE, putting individual and collective health at risk (Lázaro‐Pérez et al., 2020). In the face of a shortage of protective gear, the Spanish government purchased PPE from China for distribution throughout the country (Ministerio de Sanidad, 2020). This strategy was modified due to distribution problems that left some geographic areas short‐handed and was ultimately delegated to the regional governments (García, 2020, March 24). The failure to supply PPE had direct consequences for healthcare professionals, who faced the risk of contagion whenever they came into direct contact with people with COVID‐19 (Zhan et al., 2020). The shortage of PPE has been identified as a possible cause of contagion between healthcare professionals and patients (Suárez‐García et al., 2020).

Studies about experiences and perceptions of healthcare professionals who care for COVID‐19 patients are beginning to emerge. In a qualitative study, Sun et al. (2020) finds that Chinese nurses who cared for hospitalised COVID‐19 patients had both positive and negative emotions surrounding this experience. Zheng et al. (2021) suggest implementing mental health promotion programmes to improve the well‐being of nurses. Deliktas Demirci et al. (2021) qualitative study shows that staff shortages, scarce PPE and a lack of organisational support resulted in stress for nurses working on COVID‐19 wards. Tort‐Nasarre et al. (2021) show that nurses responded to organisational during the COVID‐19 pandemic using improvisation, adaptation, and learning strategies. Pérez‐Raya et al. (2021) offer a quantitative study of the impact of COVID‐19 on Spanish nurses, concluding that better planning is necessary to ensure safe care for patients and personal safety for nurses. The contribution of our study is to use qualitative methods to understand nurses’ experiences and perceptions of the risk of the contagion linked to the shortage of PPE during the first wave of the pandemic, whose explosive start strained health systems around the globe.

## 3 THE STUDY

### 3.1 Aim

Explore nurses’ experiences and perception of risk regarding the use of PPE during the first wave of the COVID‐19 pandemic in Spain.

### 3.2 Design

We conducted a qualitative interview‐based study, using Rapid Research Evaluation and Appraisal (RREAL, Vindrola‐Padros et al., 2020). RREAL makes it possible to obtain and analyse data in a short time (Beebe, 1995; Green & Thorogood, 2013). We were able to begin data analysis 3 weeks after the first interview was conducted.

### 3.3 Participants

The study population was nurses in Spain who were active during the first wave of the COVID‐19 pandemic, from 14 March to 4 May 2020. We used intentional sampling (Morse & Field, 1995) and identified potential participants using the snowball technique. The inclusion criterion was being a nurse, nursing supervisor or nursing director during the first wave of the pandemic. Participants included men and women between age 23 and age 61. We chose participants from different areas of nursing and geographic areas to obtain as diverse a sample as possible (Table 1).

**TABLE 1:** Sociodemographic characteristics of participants

	Nurse	Nursing supervisor	Nursing director
Sex			
Male/Female	19/5	2/1	2
Age			
23 to 29 years old	8	0	0
30 to 49 years old	10	2	1
50 to 62 years old	6	1	1
Region			
Catalonia	15	3	2
Madrid	3		
Basque Country	3		
Extremadura	2		
Cantabria	1		
Type of health centre			
Hospital	15	3	2
Field hospital	1		
Home emergencies	1		
Primary care	5		
Nursing home	2		

Authors MRL, GTN, MSM, EVS drew on their network of professional contacts to identify the initial participants, who were contacted by e‐mail and invited to participate. Participants who responded and accepted our invitation received an information sheet and an informed consent document that include details about the purpose design, expectations, risks, and benefits of the study. After participants signed the informed consent document, we arranged a time for the interview. The interviews were conducted in May 2020 using videoconferencing, given the stay‐at‐home order. We used Skype or Zoom and recorded the interviews.

### 3.4 Data collection

Three team researchers (MRL, GTN, EVS) conducted the interviews, until reaching data saturation with 29 interviews. We defined saturation as the point at which no new information was indentified from interview responses (Fush & Ness, 2015). Interviews lasted between 30–45 min and two questions were asked: What is your perception of the supply of protective equipment during the COVID‐19 pandemic? What was your experience of caring for patients during the pandemic? Due to the sensitive nature of the questions asked in this study, survey respondents were assured that raw data would remain confidential and would not be shared. The interviews were audio recorded, and the interviewers took notes and wrote memos. Both the notes and the memos were used in the RREAL process.

### 3.5 Ethical considerations

The study and the informed consent process were approved by the bioethics committee of the host university (Exp. 5184). The voice recordings and transcripts are stored in encrypted files to which only the principal investigator has access. We protected the confidentiality of participants by substituting real names with codes. The confidentiality protocol does not allow us to re‐contact participants, but the informed consent document they received contains a link where they can find the study results.

### 3.6 Data analysis

The data were anonymised and transcribed. Guided by our research questions, we analysed the most frequent topics that we identified in the interviews (see Gale et al., 2013; Smith & Firth, 2011). We followed Braun and Clarke's approach to thematic analysis (2014). We used the Dedoose® platform and we identified meaning units related to the research objectives and grouped them into sub‐themes and themes. We identified and reported patterns from the data and arranged them systematically to shed light on the research questions, while remaining profoundly respectful with the perspective of the participants (Colorafi & Evans, 2016) (see Table 2).

**TABLE 2:** Phases of thematic analysis according to Braun and Clarke (2014)

Phases	Description	Collaborators
1	Familiarisation with the data: listened to recordings, transcribed recordings, anonymisation and transcription and entering transcripts into Dedoose	MRL, GTN, BA, MSM, EVS, DM, PGE
2	Entering codes. Segmentation of meaning units within the transcribed interviews. Definition of sub‐themes and identification of relationships between sub‐themes	MRL, GTN, BA, MSM, EVS
3	The inclusion of meaning units in each sub‐theme was discussed until a consensus was reached. Grouping of meaning units into sub‐themes	MRL, GTN, BA
4	A glossary was created with the description of each sub‐theme	MRL, GTN, BA, PGE
5	All sub‐themes were reviewed, relationships between sub‐themes were identified, and they were grouped into two themes	MRL, GTN, BA, DM, PGE
6	The final report was written	MRL, GTN, BA, DM, PGE

We completed the Consolidated Criteria for Reporting Qualitative Research (COREQ), a 32‐item checklist for interviews and focus groups used to verify the quality of a study’s methods (Tong et al., 2007) (Appendix S1).

### 3.7 Rigour

Credibility, transferability, dependability, and confirmability ensure the trustworthiness of qualitative research (Polit & Beck, 2016). To ensure trustworthiness in the study, the data were discussed by MRL, GTN, BA, DM, and PGE until we reached consensus. The triangulation of observers ensures the consistency and strength of the results and therefore their trustworthiness. The interviewers carefully recorded their impressions and perceptions during the interviews and they shared these ideas with the rest of the team and used them to reflect on the beliefs, values and personal interests that could influence the research (see The University of Auckland, 2020).

### 3.8 Research team and reflexivity

The research team was made up of nurses and anthropologists. The anthropologists and three of the nurses hold Ph.Ds. and have experience with qualitative research. The external perspective of anthropologists in the health context has added strength to the analysis. Reflexivity is important in ensuring that researchers reach an accurate interpretation of the data. In this sense, team members have been aware of the necessity of maintaining distance from their own nursing practice and have counted on the perspective of the anthropologists, which has contributed to identifying the influence of their own practice on nurses during the analysis process.

## 4 FINDINGS

Two themes and 11 sub‐themes were identified related to the aim of the study (Table 3).

**TABLE 3:** Structure of themes and sub‐themes

Themes	Sub‐themes
1. Experiences with personal protective equipment	1.1 Scarcity 1.2 Inequality 1.3 Reutilisation 1.4 Self‐protection 1.5 Delegation of responsibility 1.6 Gap between protocols and reality
2. Perceptions of the risk of contagion	2.1 Lack of credibility 2.2 Lack of trust 2.3 Lack of support 2.4 Meeting subjective needs

### 4.1 Experiences related to personal protective equipment

In the first wave of the pandemic, nurses had different experiences related to PPE that were unprecedented.

#### Scarcity

Most participants faced a sudden need for PPE and experienced a shortage thereof. Some reported having access to expired, unused PPE that had been saved from the EVD pandemic.

We didn’t have the necessary material, there weren’t enough masks, we had PPE from Ebola that had been stored for four or five years. It was all a little bit hand to mouth. (P11 nurse)

Some nurses understood this as a consequence of the lack of foresight in the organisation. The sharp increase in demand for care for COVID‐19 patients and the logistical improvisation used to meet it explain the shortages.

I think that the political strategies and the managements of the centres have improvised because they had no other choice. […] We’ve had moments that we’ve adapted to what we had, to go out to battle. (P1 nurse)

Health centres improvised quickly to increase the number of beds, sometimes setting up field hospitals.

It’s logical that at first there was a lack of material because Ward 7 opened overnight: soldiers assembling beds, we too, removing plastic wrap, organising the warehouse, volunteers helping: suddenly from one day to the next 500 beds were assembled. But of course, there were a lot of nursing stations, it was 10 I think, and it's normal that material was missing, but it gradually came to us. (P18 nurse)

#### Inequality

The distribution of PPE was not only insufficient, but also uneven. Differences in access to the equipment occurred between departments, between professional categories and even between shifts. Some participants who worked in areas where PPE was lacking observed that healthcare professionals from other areas had all the material they needed.

You see that they come from the health service to do some tests in the nursing home or whatever and […] obviously, it’s nice to see how they dress, undress, all new, they take it off, they don’t touch anything. It’s a little sad, in that sense, that some have so much and others so little. (P23 nurse)

Some health centres differentiated between professional groups. According to the participants, physicians had the necessary equipment, training, and information, but nurses did not. These resources came late, if at all.

We have encountered the lack of organisation starting from the top level. I mean higher up… doctors were trained and each one received protocols, but no one gave me anything. […] The only thing is that someone came here to tell us how to remove our personal protective equipment, but they came when we had been working in the unit for three weeks already. (P10 nurse)

#### Reutilisation

In non‐specialist areas of hospitals, primary care facilities and nursing homes, PPE was reused more than in other areas. The life of masks was extended beyond manufacturers’ recommendations.

I would sometimes go out sweaty, with my whole mask wet, dripping, and wanting to change my mask and they [the supervisor] would say, ‘No! It has to last for a week’! And once a supervisor said, ‘If you all keep going like this’, she said, ‘next week you’re going in with only a surgical mask and the week after you’re going in with your hand here’ [makes the gesture of covering her mouth with her hand] (silence). (P3 nurse)

Participants who worked in units that had both patients with COVID‐19 and other patients faced an added difficulty. They were forced to keep track of which equipment they had used in which room, to avoid spreading the infection to patients who were not infected with COVID‐19.

It was chaos because at night there are three of us: me and the two nurse’s aides. We had to reuse the equipment to go into the rooms. We had to be careful not to go with the positive [gear] to the other rooms, but for suspected cases it was impossible. You couldn’t manage. (P8 nurse)

The lack of PPE was so severe that the only possible way to avoid running out was to wash, disinfect, and reuse it.

I mean, it's just that our case has been […] reusing PPE. I understand it's done because there's no equipment, […] but I think they still don't really know the importance of having to change [your equipment]. I mean, I have to take off a coverall and I have to spray it with a spray and pray it's clean, so that when I put it on in 3 hours, I don’t get infected. (P23 nurse)

Some participants reported that nurses organised themselves to disinfect and reutilise PPE.

But we still have one [PPE] for you, for as long as it lasts. We have a dirty area. We put it in there, we hang it up, we treat it with bleach diluted in water. We move it to an intermediate area, and we go over it with an ozone machine. And then we take it to a clean area, but it’s still the same [gear] with several disinfection processes. (P14 nurse)

#### Self‐protection

The nurses felt unprotected, and fear of contagion led them to use materials they had within reach, without knowing whether it really improved their safety.

We've suffered a lot over equipment. […] Looking for tutorials on how to make gowns out of garbage bags. The cleaning woman giving us large garbage bags. A midwife who knows a lot about sewing, making tutorials on how to do it. One [nurse] who has a neighbour whose husband works in China giving her masks. We had one and we kept it. My mother making cloth hats for all my co‐workers. (P2 nurse)

Participants also reported having to use expired masks, which was better than not having a mask at all, even though they didn't offer the same guarantees as unexpired masks.

We had to use plastic and make our own PPEs with rain jackets, with diving goggles. The masks were not FFP2. We had six for the whole nursing home and they were the ones from H1N1, from 2012, I think, that were super out of date. (P14 nurse)

#### Monitoring

According to the participants, the scarcity of protective gear and some cases of misuse had implications for management. The supply of PPE had to be monitored, and nurses had to justify their need for it to be provisioned. Supervising nurses distributed equipment to nurses according to the procedures they had to carry out.

[…] If I had to go inside a room, inside an operating room in this case, to do a pump that was beeping at me or change an IV… I didn't need all the equipment—the waterproof gown, the gloves […] I could go in with a double glove or a simpler gown and respiratory protection […] But if I have to go in and put in an IV, or manipulate the airway, obviously we are the first ones who know we have to be protected. And the truth is, there are times when we didn’t manage to handle this. […] We had to ration them very well. There was even a kind of conflict. (P21 nursing supervisor)

Participants who no longer had direct access to PPE (as they had enjoyed before the pandemic) had to request gear from their superiors.

In terms of the treatment that we [healthcare] professionals received by the hospital administrators, well… little [protective] equipment, you had to be asking for the equipment. (P12 nurse)

#### Delegation of responsibility

Responsibility for rationing and monitoring the equipment lay mostly with mid‐ranking nursing supervisors. Centre administrators delegated this responsibility to nursing supervisors, who found themselves in the difficult position of trying to protect their nurses while also minimising the risk of running out of PPE.

On the one hand, protecting their staff and, on the other hand, not running out of gear. And management has often delegated that responsibility to mid‐ranking positions (P25 nurse).

Some participants who were supervisors reported that PPE was used too quickly if they didn't administer it carefully.

First thing in the morning, I distribute the FFP2s. […] Because if you leave a box out, in five minutes it’s not there. Everything disappears. […] Obviously, you have to have enough for everyone, but not misuse it… (P21 nursing supervisor)

#### Gap between protocols and reality

Participants agreed that there was a gap between the protocols surrounding the use of PPE and the reality of the available equipment.

Some supervisors reported that they were unable to implement protocols rigorously because of the lack of PPE.

Because they can't say 50 protocols […] perfectly describing what to do with PPE, and then not have PPE. […] I try to make people respect the protocol as much as possible, I'll make use of what I have, right? And I'll adapt according to the general conditions or common sense in many things. But if you're telling me to put on, to throw away the FFP2 every time or the FFP3. […] Because protocol tells you that you have to throw it away every time you go in, but if you don't have [enough], you can't throw it away every time. (P21 nursing supervisor)

Several participants pointed out that even though they had received training on the proper use of PPE, they didn't receive the equipment that had been used in the training sessions.

During the month of February at the health department they were posting videos of how to put on and take off a PPE. And when we needed them there weren’t any [roars with laughter]. It’s outrageous. We've been watching videos for three weeks, and now, where are they? I take it off like this [pantomimes the action of taking off PPE], and now, where is it? There's nothing. (P2 nurse)

### 4.2 Perceptions of the risk of contagion

During the first wave of the pandemic many participants were fully aware that they were working in conditions that did not guarantee their personal safety from contagion. Four factors influenced participants’ perception of risk of contagion from SARS‐CoV‐2 during the first wave of the pandemic.

#### Lack of credibility

The lack of clear and truthful guidelines about the risk of SARS‐CoV‐2 contagion and how to protect oneself against the virus discredited public management and hospital administrations.

I follow quite a few healthcare professionals on social media who talked about how odd the ministry's recommendations were on which equipment to use, which seemed to be decreasing. At first, they would tell us FFP3 masks and then they were going down. That is, because there wasn’t enough gear, they lowered their PPE recommendations. This generated a bit of a lack of credibility. Then when equipment arrived from China, it was written in Chinese so we understood nothing, but the numbers written as FFP5. When more equipment started to arrive, we had to ask less, but of course, then comes the news of defective equipment, etc. You're putting the [healthcare] professionals you have to take care of at considerable risk. (P12 nurse)

The protocols of each centre also varied and often failed to cover all of the situations that nurses encountered.

They haven’t stipulated well what PPE we should wear when we go to work in a dirty area. It depends a bit on the judgement of the person and on the equipment that’s available that day. It depends on how the wind blows. It’s very clear when you go to treat a respiratory patient how you should dress. Now if you’re going to work in areas that are potentially contaminated, but you’re not going directly to a COVID‐19 patient, there’s not a protocol for protecting yourself. Sometimes the recommendations are even contradictory: there are areas that are very well defined, others not at all… This generates confusion since in the end you don’t know what you should do in each case. (P29 nurse)

#### Lack of trust

The scarcity of PPE led nurses to mistrust centre administrators and even their peers. Participants interpreted administrators’ concern about saving equipment as a lack of interest in their safety. This lack of trust appeared in some participants who perceived that the workers who were responsible for distributing PPE wore better quality equipment than that which they distributed.

It's true that we got a little angry because the people who handed out the equipment were the emergency department people […]. It was therefore a little suspicious that they brought us those hard masks, but they came wearing their 3M mask. At that moment, we wondered why they had those and we were brought the hard egg‐shaped masks. (P18 nurse).

*Some participants reported that the information that they received was not reliable*, *and they feared that administrators were withholding information about the risk of contagion to justify distributing less PPE*.

I understand it's a changing situation, but we could have been more careful with information overload. Sometimes they would also try to take advantage of new situations to avoid sending you more PPE because they said there was no danger anymore, etc. It’s much more difficult to work this way because we were working from [a perspective of] generalised mistrust, even among co‐workers. […] That makes people think they're more worried about saving [equipment] than about protecting people. (P16 nurse)

#### Lack of support

Some participants felt a lack of support from their supervisors that added to their feelings of vulnerability and fear.

I haven’t been afraid, not personally afraid… I mean, it’s my job. I chose to be a nurse, and I’m here for whatever comes. But I have felt unprotected by the system and by my superiors. At no time have I heard that comfort of: ‘Let’s go girls, one day less! It’s okay, we’re going to try to do it together! What do you need? What are you missing’? No, it was all fear, that the gear shouldn’t be used up, that there aren’t masks, that there aren’t PPE. (P3 nurse)

Sometimes, the lack of support led nurses to question whether they wanted to continue in their jobs.

There has been a lot of… a feeling, a perception of a lack of safety for the worker. And even at sometimes people have even been forced to wonder to what extent they wanted to keep providing that care, because they haven’t felt supported or backed up. (P14 nurse)

#### Meeting subjective needs

Lack of knowledge about the virus and contagion pathways raised a lot of concern among nurses. Nursing supervisors had protocols for distributing PPE according to objective criteria. However, the nurses’ subjective perception of risk generated fear that needed to be managed. Mid‐ranking nursing surpervisors had to handle these subjective emotional needs of their nurses, showing comprehension of their experiences and perceptions.

[A lack of PPE] hasn't occurred at the hospital. It was more [a question of] FFP2 or FFP3. ‘That one [nurse] is wearing an FFPx. Why aren’t I’? And you had to be with them a little bit and explain the difference. Taking protocols and keeping up to date with protocols coming out of occupational health, so that people saw that we're already protected. And it depends on the type of patient you have. So, ‘She has to be protected a little more, with a surgical gown’, and you had to be very on top of the situation. There was fear… It’s true that the needs and perceptions of the staff, be they COVID dirty rooms, COVID clean rooms. The perception is that they were under‐protected, and they demanded this protection without having objective criteria. It was based more on subjective criteria. […] ‘Do you feel safer if you're wearing this? Well, here, no problem’! They [supervisors] end up understanding that it’s not worth it to argue and continually put the regulations on the table. But rather give more weight to people’s subjective judgement. ‘Do you feel better? Well, here, go ahead’! (P28 nursing supervisor).

The restrictions on PPE caused concern, tension, and fear among nurses, who in turn felt unable to make changes to ameliorate the situation.

On the one hand, you wanted to help, because your co‐worker comes to demand a series of protective items, which sometimes there weren’t any or we didn’t have approval from the administration to hand them out. Protocols are very restrictive for using PPE. So, we’ve experienced a lot of tension, with a lot of fear on the part of all [healthcare] professionals, and we have found ourselves with little wiggle room for improving this situation. (P25 nurse)

## 5 DISCUSSION

Our results reveal the participants’ experiences with PPE as well as the factors that influenced their perception of risk during the first wave of the COVID‐19 pandemic.

### 5.1 Experiences related to personal protective equipment

These results show the severity of the shortage of PPE that affected nurses and other healthcare professionals during the first wave of the pandemic in Spain (Suárez‐García et al., 2020). This shortage was experienced around the world (Iqbal & Chaudhuri, 2020), contributing to the fact that nurses are the healthcare professionals that experienced the highest incidence of infection (Bandyopadhyay et al., 2020). A scarcity of PPE also occurred during the EVD pandemic in West Africa (Adongo et al., 2017). Additionally, Australian nurses pointed to a lack of PPE to care for people infected with H1N1 influenza in 2009 (Corley et al., 2010). Our results show that, despite these recent experiences of PPE shortages around the world, shortages were not prevented at the beginning of the COVID‐19 pandemic. Moreover, we show how nurses experienced these shortages. Notably, in many instances they reported that the unexpected explosion of the pandemic had prevented institutions from preparing.

Our finding that healthcare professionals had unequal access to PPE during the first wave of the pandemic is consistent with a study by Jia et al. (2020), which shows that hospital nurses in Shandong were less protected than doctors. Different degrees of access to PPE among different health units have also been found in the UK (Hoernke et al., 2021). PPE shortages were most notable in care homes, community health facilities and general practice. The unavailability of PPE at the onset of the pandemic had consequences for the risk of contagion among healthcare professionals, with important implications for their health and the risk of transmission to healthy patients (Suárez‐García et al., 2020). According to our study, inequality in access to equipment produced differences between departments and even between co‐workers, leading to discomfort among co‐workers and increased perception of risk.

Reusing PPE was also linked to the perception of risk, according to our results. Non‐specialised services in hospitals, primary care facilities and nursing homes faced the most scarcity of PPE, and these areas identified more initiatives by nurses to reutilise gear. This result is consistent with those of Hoernke et al. (2020), and there are also precedents of reuse during the H1N1 influenza pandemic in Australia, which led to increased perception of risk of contagion among nurses (Corley et al., 2010). The reutilisation of material should be performed according to the manufacturer's instructions, but it is still unknown whether reuse is recommended in the case of COVID‐19 (Bessesen et al., 2015; De Perio et al., 2020). These reuse initiatives were taken without knowledge of the manufacturer's instructions, and they were attempts to improve protection, even though efficacy was not guaranteed. These findings extend those by Liu et al. (2020), which show that doctors and nurses in China reused PPE. At the same time, many participants engaged in self‐protection using non‐approved do‐it‐yourself materials, as was common throughout Spain. This result, consistent with Hoernke et al. (2020), shows that nurses took the initiative to protect themselves and continue caring for people who were sick with COVID‐19. These results indicate that when nurses felt vulnerable and lacked knowledge about the risk of contagion, they sought out protective gear that could alleviate their sense of risk.

Healthcare professionals knew that level 3 of biosecurity protection had been activated in China, following WHO recommendations (Zhan et al., 2020). Spanish health institutions had difficulty supplying healthcare professionals with PPE (Suárez‐García et al., 2020). This led to increased monitoring of PPE, which meant a change in the typical working dynamics of resource managers and nurses. Many health centres administrations delegated to mid‐ranking nursing supervisors the management of PPE and limited the access of general nursing staff. Mid‐ranking nurses rationed PPE to avoid running out. Our results indicate that, for mid‐level nursing supervisors, taking responsibility for monitoring PPE was difficult to combine with ensuring the protection of nurses, given the shortage of equipment. Prior to the pandemic, nurses in Spain were entrusted with distributing PPE among themselves as necessary to care for patients. During the first wave, mid‐ranking supervisors became responsible for this task, leading some participants to sense that supervisors were more concerned with maintaining stocks of PPE than with protecting nurses’ health.

Protocols on the use of protective gear in Spain were updated frequently, as information about SARS‐CoV‐2 and its transmission pathways became known. These results highlight that a gap developed between safety protocols and the availability of PPE. A similar gap between protocols and availability occurred during the H1N1 pandemic in Australia. The difference between protocols and clinical reality caused confusion and jeopardised the protection of nurses due to a lack of knowledge about the virus (Corley et al., 2010). We highlight that the scarcity of PPE caused not only confusion, but also fear, among our participants.

### 5.2 Perceptions of the risk of contagion

Our results indicate that the lack of credibility of health administrators resulted from the lack of clarity in protocols and veracity about the risk of SARS‐CoV‐2 infection and how to prevent it. The lack of specific protocols and the existence of contradictions between different protocols led to confusion and a feeling of insecurity among the participants. Lázaro‐Pérez et al. (2020) remark that the lack of clarity affected the well‐being of healthcare professionals and patients. We see a precedent in the confusion that occurred among emergency room nurses in Hong Kong when faced with information overload during the human swine influenza epidemic of 2009 (Lam & Hung, 2013). Lam and Hung's study showed that, ironically, excess information led to confusion and increased risk of contagion. We have shown that, in addition to impairing the credibility of health administrators among the nurses they employ, confusing protocols accentuated the sense of risk among participants.

Participants felt a lack of trust in management when it seemed that management was more concerned about rationing gear than about their safety. Mistrust also occurred among healthcare professionals of the English National Health Service because of poor handling of the lack of PPE during the pandemic (Iqbal & Chaudhuri, 2020). Pincha Baduge et al. (2017) have written about the perception of risk among emergency nurses in Australia who were preparing for potential cases of EVD (which in fact never arrived). The authors conclude that when the organisation provided the appropriate gear, with well‐defined processes and protocols tailored to the needs of the department, nurses trusted their managers. Our results indicate the importance of prioritising the care and protection of nurses to preserve their confidence in management.

During the first wave of the pandemic, nurses worked in precarious conditions, with few safety protections, resulting in anxiety. Mental health teams were deployed to provide support to healthcare professionals, but this help was distributed unevenly across institutions and geographic areas (Lázaro‐Pérez et al., 2020). The precedent of China revealed the emotional impact the pandemic had on healthcare professionals and the need for health institutions to support their well‐being (Liu et al., 2020). We stress that, although our participants had access to psychologists, they missed the support and care of their superiors during the workday. They felt vulnerable and reported that they needed their supervisors to support them and recognise their effort. Support from management is key for increasing nurses’ sense of security and decreasing their perception of risk.

Lack of knowledge about how the virus spreads is an added stress that substantially increases fear among healthcare professionals (Liu et al., 2020). Fear of contagion decreases when there is an adequate supply of adequate protective gear (Liu et al., 2020), which is the responsibility of institutions in pandemics (Lam & Hung, 2013). Fear can be mitigated by providing training on prevention measures that provide greater safety and lessen psychological pressure (Mo et al., 2020). We have shown that meeting nurses’ subjective needs related to fear of contagion led to fewer disagreements among nurses over the scarcity and distribution of protective gear.

According to Watkins et al. (2006) to improve infection control strategies, consideration should be given to the influence of individual risk perception (see Gerbert et al., 1988, described in ‘Background’), as well as the culture of the organisation and infection control policies involved in nursing practice. We have identified lack of credibility, lack of trust and lack of support as factors influencing participants’ perception of risk. Meeting nurses’ subjective needs as they face the risk of contagion often lay with mid‐ranking nursing supervisors. Awareness, acceptance, and comprehension of the subjective experience of risk helped some nursing supervisors alleviate nurses’ fear and discomfort.

### 5.3 Limitations and future directions

The qualitative design has allowed us to explore the experiences and perceptions of nurses from different departments and different geographical areas but does not make it possible to generalise the results to all nurses.

Another limitation is that it has been difficult to distance ourselves from the health crisis we were studying because it is severely affecting the entire population. All team members have been affected by the pandemic as users of health services, family members of a person who has been sickened by or died from COVID‐19 and/or nurses working in frontline health care in Spain. We have had to be conscious of the individual situation of each team member.

One possible line of future research would be to further investigate nurses’ perception of risk with respect to COVID‐19 and PPE at another stage of the pandemic. The abruptness of the onset and the severity of the pandemic decisively influenced the work of nurses, their protection at work and their perception of risk. It would therefore be interesting to know how experiences and perceptions change as the pandemic progresses. This line of inquiry could also be extrapolated to future health crises or epidemics that are foreseeable due to habitat loss among many animal species and global hyper‐connectivity.

## 6 CONCLUSION

The scarcity of PPE and inequality in their distribution led participants to take initiatives to feel more protected and maintain their commitment to care for people infected with COVID‐19. Participants who were mid‐ranking supervisors were caught between the responsibility of monitoring and rationing PPE and providing the necessary protection to nurses. The disjuncture between protocols and the available supply of PPE caused fear. Lack of credibility, lack of trust and lack of support influenced participants’ perception of the risk of contagion, and mid‐ranking supervisors were responsible for trying to alleviate it.

## 7 IMPLICATIONS FOR CLINICAL PRACTICE

Health centre management must provide the necessary PPE to nurses in health crises. The lack of sufficient protective gear leads nurses to seek alternative forms of protection, whose use may increase the risk of contagion. A lack of trust in management may increase nurses’ perception of risk. Understanding the factors involved in risk perception can be helpful to decision‐makers who help protect nurses in clinical practice. These results can help administrators and policymakers because they point to the need for nurses to feel that their departments and centres look after their safety at work. Of course, the most important thing that health centres can do is provide the appropriate protection to nurses. When this is impossible—as in the first wave of the COVID‐19 pandemic—transparent communication and emotional support may contribute to improving nurses’ well‐being in the face of risk.

## CONFLICT OF INTEREST

The authors declare that they have no conflict of interests.

## AUTHOR CONTRIBUTIONS

All authors were substantially involved in the design of the study, and development of research and interview questions. MRL, GTN, BA, MSM and EVS completed and transcribed the interviews, as well as initial analysis and coding of data. MRL, GTN, BA, DM and PGE discussed the codings throughout the analysis, and were extensively involved in revising and finalising the themes. MRL wrote the paper and prepared the manuscript for journal submission; all authors contributed substantially to editing the draft. All authors read and approved the final manuscript.

## Supporting information

Supplementary Material
