# Supplementary material for: Spanish nurses’ experiences with personal protective equipment and perceptions of risk of contagion from COVID‐19: A qualitative rapid appraisal
Source: J Clin Nurs. 2021 Sep 15;31(15-16):2154–66. doi: 10.1111/jocn.16031 (PMC8662180; doi:10.1111/jocn.16031)
Supplement: Supplementary file 1 — Supplementary Material [file JOCN-31-2154-s001.docx]

Appendix S1. - Consolidated criteria for reporting qualitative studies (COREQ): 32-item checklist

| Guide questions/description | Page/Answers |
| --- | --- |
| **Domain 1: Research team and reflexivity** | |
| *Personal Characteristics* | |
| 1. Interviewer/facilitator-Which author/s conducted the interview or focus group? | Page 6 |
| 2. Credentials-What were the researcher’s credentials? E.g. PhD, MD | 5 PhD and 2 PhD candidate |
| 3. Occupation-What was their occupation at the time of the study? | 2 nurses in COVID-19 mission, 2 nurses in COVID-19 mission and university professors, 1 nurse and anthropologist university professor, 2 anthropologist university professors |
| 4. Gender-Was the researcher male or female? | Both male and female |
| 5. Experience and training-What experience or training did the researcher have? | The researchers have participated in qualitative studies before. |
| *Relationship with participants* | |
| 6. Relationship established-Was a relationship established prior to study commencement? | Page 6. Previous relationship with initials contacts |
| 7. Participant knowledge of the Interviewer-What did the participants know about the researcher? e.g. personal goals, reasons for doing the research | Page 6. Information were provided in recruiting. |
| 8. Interviewer characteristics-What characteristics were reported about the interviewer/facilitator? e.g. Bias, assumptions, reasons and interests in the research topic | Page 7. Researchers carefully ensured rigor. |
| **Domain 2: study design** | |
| *Theoretical framework* | |
| 9. Methodological orientation and Theory-What methodological orientation was stated to underpin the study? e.g. grounded theory, discourse analysis, ethnography, , content analysis | Page 6. Rapid Research Evaluation and Appraisal (RREAL) |
| *Participant selection* | |
| 10. Sampling-How were participants selected? e.g. purposive, convenience, consecutive, snowball | Page 6. Intentional sampling using snowball technique. |
| 11. Method of approach-How were participants approached? e.g. face-to-face, telephone, mail, email | Page 6. Videoconferencing interviews |
| 12. Sample size-How many participants were in the study? | Page 6. 29 participants |
| 13. Non-participation-How many people refused to participate or dropped out? Reasons? | Page 6 |
| *Setting* | |
| 14. Setting of data collection Where was the data collected? e.g. home, clinic, workplace | Page 6 |
| 15. Presence of non-participants Was anyone else present besides the participants and researchers? | Page 6. Participants were interviewed individually. |
| 16. Description of sample-What are the important characteristics of the sample? e.g. demographic data, date | Page 6 and Table 1. |
| *Data collection* | |
| 17. Interview guide-Were questions, prompts, guides provided by the authors? Was it pilot tested? | Page 6. Semi-structured interviews |
| 18. Repeat interviews-Were repeat interviews carried out? If yes, how many? | No repeat interview. |
| 19. Audio/visual recording-Did the research use audio or visual recording to collect the data? | Page 6. Audio record. |
| 20. Field note- Were field notes made during and/or after the interview or focus group? | Page 6-7 |
| 21. Duration What was the duration of the interviews or focus group? | Page 6. 30–45 minutes. |
| 22. Data saturation-Was data saturation discussed? | Page 6 |
| 23. Transcripts returned-Were transcripts returned to participants for comment and/or correction? | Page 7. The confidentiality protocol does not allow us to re-contact participants. |
| **Domain 3: analysis and findings** | |
| *Data analysis* | |
| 24. Number of data coders-How many data coders coded the data? | Page 7. Table 2. |
| 25. Description of the coding tree-Did authors provide a description of the coding tree? | Page 7. Table 4. |
| 26. Derivation of themes-Were themes identified in advance or derived from the data? | Page 7 |
| 27. Software What software, if applicable, was used to manage the data? | Page 7. Dedoose |
| 28. Participant checkin- Did participants provide feedback on the findings? | Page 7 |
| *Reporting* | |
| 29. Quotations presented-Were participant quotations presented to illustrate the themes / findings? Was each quotation identified? e.g. participant number | Page 8-17 |
| 30. Data and findings consistent-Was there consistency between the data presented and the findings? | Page 8-17 |
| 31. Clarity of major themes Were major themes clearly presented in the findings? | Page 8-17 |
| 32. Clarity of minor themes Is there a description of diverse cases or discussion of minor themes? | Page 8-17 |
